# Elevated serum calprotectin levels in major depressive disorder: no evidence of association with S100A9 rs3014866 polymorphism

**DOI:** 10.1186/s12888-026-08161-3

**Published:** 2026-05-13

**Authors:** Furkan Akbas, Ozgur Baykan, Ayla Solmaz Avcikurt, Hayriye Baykan

**Affiliations:** 1 Department of Psychiatry, Balıkesir University Faculty of Medicine, Balıkesir, Turkey; 2 Department of Medical Biochemistry, Balıkesir University Faculty of Medicine, Balıkesir, Turkey; 3 Department of Medical Genetics, Balıkesir University Faculty of Medicine, Balıkesir, Turkey

**Keywords:** Calprotectin, Major depressive disorder, rs3014866, Genetic polymorphism

## Abstract

**Background:**

Major depressive disorder (MDD) has been increasingly associated with low-grade inflammation, with neutrophils playing a central role. Calprotectin is a mainly neutrophil-derived inflammatory mediator. We investigated whether serum calprotectin levels are higher in patients with MDD than in healthy controls and whether the S100A9 rs3014866 polymorphism is associated with serum calprotectin levels and with MDD.

**Methods:**

We enrolled 66 patients with major depressive disorder (MDD) and 54 healthy controls. Depressive symptoms were assessed using the 17-item Hamilton Depression Rating Scale (HAM-D). Fasting venous blood samples were collected to measure serum calprotectin and to genotype the S100A9 rs3014866 polymorphism.

**Results:**

Serum calprotectin levels were higher in patients with MDD than in healthy controls (2.18 ± 1.33 vs. 1.05 ± 0.55 µg/mL; *p* < 0.001). Serum calprotectin levels were positively correlated with HAM-D scores among patients with MDD (rs = 0.314, *p* = 0.010). The S100A9 rs3014866 genotype distribution was in Hardy–Weinberg equilibrium. Genotype and allele frequencies did not differ significantly between patients with MDD and healthy controls, and serum calprotectin levels did not differ significantly across genotype groups under the tested inheritance models.

**Conclusions:**

These findings suggest that serum calprotectin may reflect inflammatory processes associated with MDD and that its potential role as a biomarker warrants further investigation.

## Background

Major depressive disorder (MDD) is one of the leading mental illnesses worldwide and reduces quality of life across many areas; in the Global Burden of Disease 2021, depressive disorders rank second in years lived with disability [1]. MDD is characterized by anhedonia; disturbances in sleep, appetite, psychomotor activity, and cognition; persistent depressed mood; loss of interest or pleasure; recurrent thoughts of death; and other somatic and cognitive symptoms, as defined in the Diagnostic and Statistical Manual of Mental Disorders, Fifth Edition, Text Revision (DSM-5-TR) [2].

The pathogenesis of MDD is not yet fully understood, and patients diagnosed with MDD may show considerable heterogeneity in symptom profiles, illness trajectories, treatment responses, and associated biological features [3]. Inflammation represents one dimension of this heterogeneity, and accumulating evidence implicates low-grade systemic inflammation and immune dysregulation in the pathophysiology of MDD [3–5]. This inflammatory activation is not uniformly present across patients with MDD and varies according to the marker and threshold used [4, 5]. In a meta-analysis of CRP levels, low-grade inflammation, defined as CRP > 3 mg/L, was reported in 27% of patients with depression, whereas mildly elevated CRP, defined as CRP > 1 mg/L, was observed in 58% [4]. In addition to CRP, altered levels of cytokines and immune-related markers, including IL-6, TNF-α, IL-1RA, IL-12, IL-18, and sIL-2R, have also been reported in depression, although these findings primarily reflect group-level differences and do not indicate that all patients show elevations in these markers [5]. Neutrophil-related alterations have also been reported in MDD cohorts, including higher neutrophil counts and elevated neutrophil-to-lymphocyte ratios relative to controls [6, 7]. During inflammatory activation, neutrophils release several mediators, including calprotectin (S100A8/S100A9; also known as myeloid-related protein 8/14 [MRP8/14]) [8].

Calprotectin, a heterodimeric protein of the S100 family composed of the subunits S100A8 and S100A9, binds calcium and zinc [8]. It is produced mainly by neutrophils, in which it constitutes approximately 60% of cytosolic proteins, and, to a lesser extent, by monocytes and macrophages [9]. It acts as an inflammatory marker and has antimicrobial and antiproliferative properties [9]. It facilitates the recruitment and migration of neutrophils, monocytes, and other inflammatory cells to sites of inflammation [10]. By binding to polyunsaturated fatty acids, it modulates eicosanoid-mediated responses, and disruption of this pathway has been associated with reduced neutrophil migration and cytokine release [11, 12]. The genes encoding S100A8 and S100A9 are located on chromosome 1q21 [13]. S100A9 encodes a subunit of the S100A8/A9 calprotectin complex, and genetic variation near this gene has been examined in relation to calprotectin-associated inflammatory processes [8, 13]. The rs3014866 (C > T) polymorphism is a common 5′-upstream variant near S100A9 and has been investigated in several inflammation-related conditions, including insulin resistance, type 2 diabetes, Crohn’s disease treatment response studies, ischemic stroke, and Parkinson’s disease [14–18]. In these studies, rs3014866 has been linked to circulating S100A9 levels, S100A9 expression, insulin resistance-related phenotypes, type 2 diabetes susceptibility, ischemic stroke risk, and Parkinson’s disease risk. In Crohn’s disease, rs3014866 has been included in S100A8–S100A9 haplotype analyses of infliximab response [14]. However, evidence regarding its direct influence on S100A9 transcription, protein expression, or circulating S100A8/A9 calprotectin levels remains limited. In this context, rs3014866 was selected as a candidate polymorphism to examine its potential association with serum calprotectin levels and MDD.

This study aimed to determine whether serum calprotectin levels are elevated in patients with MDD compared with healthy controls (HC) and to evaluate calprotectin as a potential clinical biomarker. We also examined whether the S100A9 rs3014866 polymorphism is associated with serum calprotectin levels and MDD.

## Methods

### Study design and setting

Between July 2023 and April 2025, we conducted an observational case–control study at the Department of Psychiatry, Balıkesir University Faculty of Medicine, Turkey. The local ethics committee approved the study (Approval No: 2023/70), and all procedures were conducted in accordance with the Declaration of Helsinki. Before enrollment, all participants received detailed information about the study and provided written informed consent.

### Participants and clinical assessments

We initially enrolled 126 individuals in the study. In accordance with predefined biomarker quality-control criteria, six participants were excluded listwise due to lipemic or hemolyzed serum samples (*n* = 4) and genotyping errors (*n* = 2). The final analytic sample, therefore, consisted of 120 participants, including 66 patients with MDD and 54 healthy controls.

Eligibility was determined during pre-enrollment screening, which included a comprehensive medical history and medication review. Inclusion criteria were age 18–65 years, a diagnosis of MDD according to DSM-5-TR, and providing written informed consent. All patients with MDD were newly diagnosed and had not received any antidepressant or other psychotropic treatment prior to enrollment and blood sampling. Before diagnostic classification, all candidates were screened for lifetime mania/hypomania and any history suggestive of treatment-emergent mood elevation to exclude bipolar disorder. Exclusion criteria included a current or past diagnosis of an organic mental disorder, schizophrenia, schizophreniform, or other psychotic disorders, pregnancy, substance use disorder, neurodegenerative disorders, an active anxiety disorder, or any uncontrolled or severe medical condition. We also excluded individuals with celiac disease, inflammatory bowel disease, other autoimmune disorders, or acute/chronic gastrointestinal inflammatory conditions, as well as those currently taking systemic corticosteroids or other immunomodulatory therapies. All participants were assessed using the Structured Clinical Interview for DSM Disorders (SCID) to confirm diagnoses and rule out additional psychiatric comorbidities. Following the interview, participants completed a sociodemographic questionnaire collecting information on age, sex, smoking status, alcohol use, body mass index (BMI), chronic medical conditions, income level, and current medication use.

In patients with MDD, depressive symptom severity was assessed using the 17-item Hamilton Depression Rating Scale (HAM-D), a clinician-rated tool with mixed 0–2 and 0–4 anchors (total score range 0–52). The Turkish validation reported Cronbach’s α of approximately 0.75, split-half reliability of approximately 0.76, test–retest reliability (r) of approximately 0.85 over 5 days, and inter-rater reliability (r) of approximately 0.87–0.98.

### Serum calprotectin measurement

After an 8–12-hour fast, all participants provided venous blood samples, which were collected into VACUSERA serum gel & clot activator tubes (Disera A.Ş., Turkey). The tubes were centrifuged at 1,500 × g for 10 min, and then serum was separated, transferred to Eppendorf tubes, and stored at − 40 °C until analysis. Just before testing, samples were gradually thawed—initially at 4 °C and then at room temperature. Serum calprotectin levels were measured in duplicate using a commercially available Human Calprotectin ELISA kit (Elabscience, Houston, TX, USA) according to the manufacturer’s instructions, and the mean of the duplicate measurements was used for analysis.

### Genotyping

Venous blood samples were collected into EDTA tubes (BD Vacutainer, USA) for DNA extraction. Genomic DNA was isolated using a Genomic DNA Purification Kit (Thermo Fisher Scientific, MA, USA) according to the manufacturer’s protocol.

The single-nucleotide polymorphism (SNP) rs3014866 (C > T) in S100A9 (chromosome 1q21.3) was selected for analysis. Genomic coordinates were GRCh38.p14: chr1:153,356,595 and GRCh37.p13: chr1:153,329,071 (RefSeq NC_000001.10:g.153329071T > C). The sequence context was CGAGGGTGTC[T/C]CTCTTGCCAA. We pre-specified rs3014866 as a biologically plausible candidate because S100A9 forms the S100A8/A9 (calprotectin) heterodimer, the circulating marker analyzed in this study.

Genotyping was performed by real-time polymerase chain reaction (PCR) using TaqMan allelic discrimination assays with predesigned probes (VIC for the wild-type allele; FAM for the variant allele) on an Applied Biosystems 7500 Real-Time PCR System (CA, USA). The PCR protocol consisted of an initial denaturation at 95 °C for 10 min, followed by 40 cycles of 95 °C for 15 s and 60 °C for 1 min. Negative controls and duplicate samples were included to ensure accuracy. Genotype calls were analyzed with Applied Biosystems 7500 Real-Time PCR Software v1.5.1 (Applied Biosystems, Foster City, CA, USA).

### Statistical analysis

Sample size calculation was performed a priori using G*Power version 3.1.9.4. Because no prior studies directly evaluating serum calprotectin levels in patients with primary MDD were available to provide an established effect-size estimate for sample-size calculation, Cohen’s conventional medium effect size was used. For comparing two independent means between two groups, with Cohen’s d = 0.50, α = 0.05, and 80% power, the minimum required sample size was calculated as 128 participants, with 64 participants in each group.

Statistical analyses were performed using SPSS version 27.0 (IBM Corp., Armonk, NY, USA) and MedCalc Statistical Software version 20.106 (MedCalc Software Ltd, Ostend, Belgium). Data distribution was evaluated using visual methods (e.g., histograms) and the Kolmogorov–Smirnov test. Differences between groups were analyzed using Student’s t-test for normally distributed variables and the Mann–Whitney U test for non-normally distributed variables. When more than two independent groups (e.g., genotype subgroups) were compared, one-way analysis of variance (ANOVA) or the Kruskal–Wallis test was used, depending on the distributional assumptions. Categorical variables were compared using the chi-square test. Genotype distributions in patients and controls were assessed for Hardy–Weinberg equilibrium, and differences in genotype and allele frequencies between groups were examined using the chi-square test. Receiver operating characteristic (ROC) curve analysis was conducted to evaluate the ability of serum calprotectin levels to discriminate between patients with MDD and healthy controls, and sensitivity and specificity were determined at the selected cutoff value. Multiple linear regression analysis was used to assess whether serum calprotectin levels were independently associated with depressive symptom severity after adjustment for relevant covariates. Associations between continuous variables were assessed using Pearson’s correlation or Spearman’s rank correlation (rs), depending on distributional characteristics. A p-value < 0.05 was considered statistically significant.

## Results

Of the 126 enrolled participants, 6 (4.8%) were excluded due to biomarker quality-control criteria (ELISA hemolysis/lipemia: HC *n* = 4; genotyping failure: HC *n* = 2), resulting in a final analytic sample of 120 participants (95.2%). There were no missing data for psychometric assessments, ELISA measurements, or rs3014866 genotypes within the analytic dataset.

The mean ages of the MDD and HC groups were 40.4 ± 13.5 and 40.2 ± 12.1 years, respectively, with no significant difference (*p* = 0.937; Table 1).

Among patients with MDD, 45.5% were male, compared to 42.6% in the HC group, indicating no statistically significant difference between the groups (*p* = 0.753; Table 1).

**Fig. 1:**
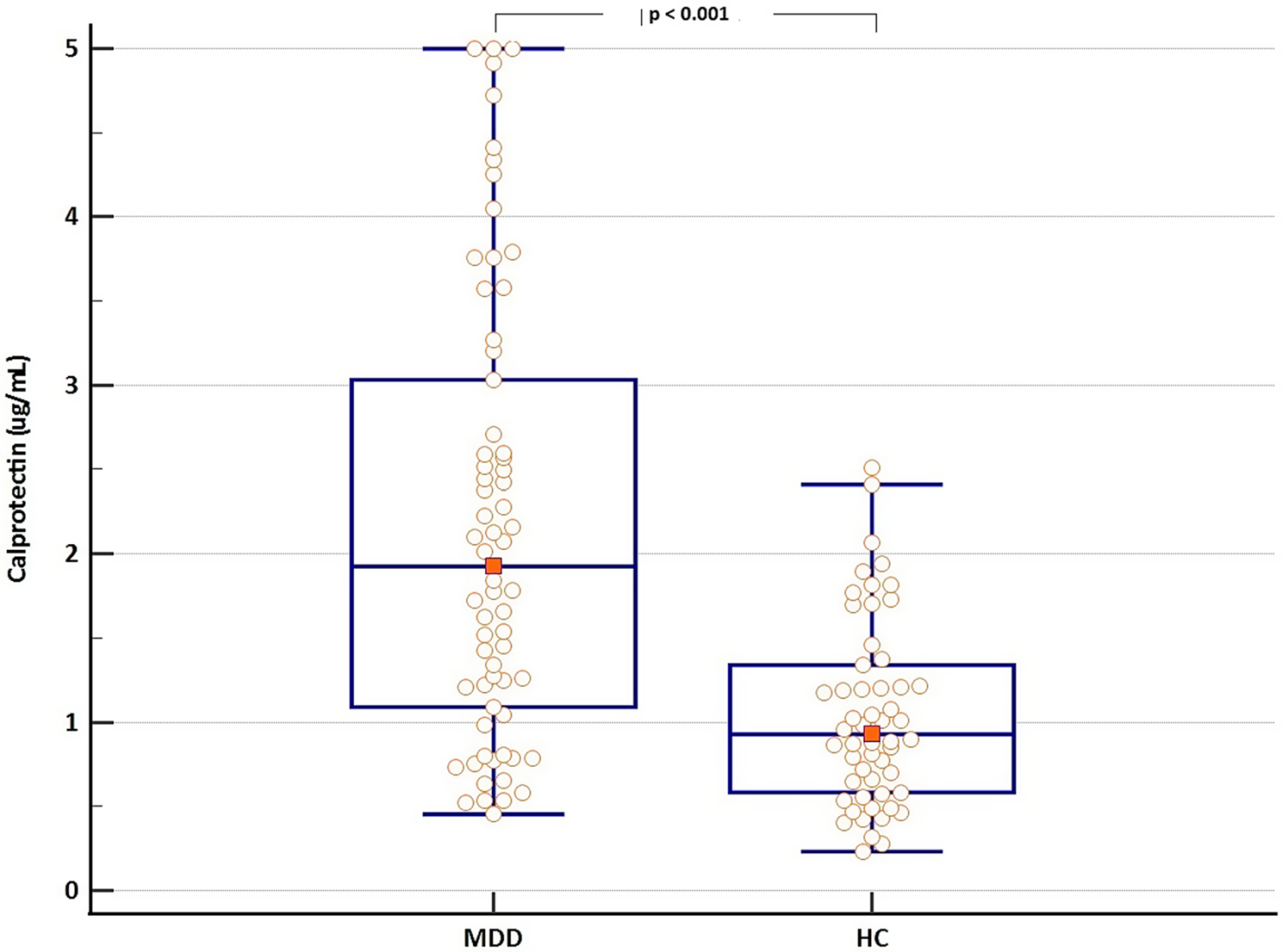
Dot plot of serum calprotectin levels in patients with MDD and healthy controls (*p* < 0.001)

The mean serum calprotectin level was 2.18 ± 1.33 µg/mL in the MDD group and 1.05 ± 0.55 µg/mL in the HC group, with a statistically significant difference (*p* < 0.001; Table 1; Fig. 1).

Mean BMI values were 26.61 ± 4.18 in the MDD group and 26.35 ± 3.71 in the HC group, with no statistically significant between-group difference (*p* = 0.721; Table 1). Across all participants, BMI was not significantly correlated with serum calprotectin levels (Spearman’s rs = − 0.166, *p* = 0.069). In subgroup analyses, no significant correlation was observed between BMI and serum calprotectin levels in the HC group (rs = − 0.144, *p* = 0.298; Table 2). In contrast, a significant negative correlation was observed in the MDD group (rs = − 0.255, *p* = 0.039; Table 2). In the regression model conducted in the MDD group, including both BMI and serum calprotectin levels as predictors of depression severity, only serum calprotectin levels were significantly associated with depression severity (B = 1.122, *p* = 0.002), while BMI was not (B = 0.046, *p* = 0.700).

The median (min–max) pack-years were 0 (0–30) in the MDD group and 0 (0–40) in the HC group, with no significant difference between the groups (*p* = 0.140; Table 1). Across all participants, smoking pack-years were not correlated with serum calprotectin levels (rs = − 0.020, *p* = 0.826).

Alcohol use was reported by 13 patients with MDD (19.7%) and 12 healthy controls (22.2%), with no significant difference between the groups (*p* = 0.735; Table 1). Serum calprotectin levels did not differ significantly between participants with and without alcohol use (*p* = 0.209).

Chronic medical conditions were reported by 13 patients with MDD and 7 healthy controls. The prevalence of at least one chronic medical condition did not differ significantly between the MDD and HC groups (13/66, 19.7% vs. 7/54, 13.0%; *p* = 0.325; Table 1). The reported chronic medical conditions included hypertension (MDD: *n* = 6; HC: *n* = 5), type 2 diabetes mellitus (MDD: *n* = 5; HC: *n* = 4), chronic obstructive pulmonary disease (MDD: *n* = 1; HC: *n* = 1), and cardiovascular disease (MDD: *n* = 3; HC: *n* = 2). These categories were not mutually exclusive, as some participants had more than one chronic condition. When the analysis was repeated after excluding participants with chronic medical conditions, serum calprotectin levels remained significantly higher in patients with MDD than in healthy controls (MDD: *n* = 53; HC: *n* = 47; *p* < 0.001).

**Table 1:** Demographic and clinical characteristics and serum calprotectin levels in patients with major depressive disorder and healthy controls

	MDD	HC	*p*
Serum calprotectin level (µg/mL, mean ± SD)	2.18 ± 1.33	1.05 ± 0.55	< 0.001
Age (mean ± SD)	40.4 ± 13.5	40.2 ± 12.1	0.937
Sex (% male)	45.5%	42.6%	0.753
BMI (kg/m², mean ± SD)	26.61 ± 4.18	26.35 ± 3.71	0.721
HAM-D score (mean ± SD)	15.83 ± 4.16	N/A	N/A
Suicidal ideation/behavior, n (%)	23 (34.8)	N/A	N/A
Smoking, pack years, median (min–max)	0 (0–30)	0 (0–40)	0.140
Alcohol use, n (%)	13 (19.7%)	12 (22.2%)	0.735
Chronic disease, n (%)	13 (19.7%)	7 (13.0%)	0.325

HC: healthy controls; MDD: major depressive disorder; BMI: body mass index; HAM-D: Hamilton Depression Rating Scale; SD: standard deviation; N/A: not applicable. HAM-D scores and suicidal ideation or behavior were evaluated only in patients with MDD

**Fig. 2:**
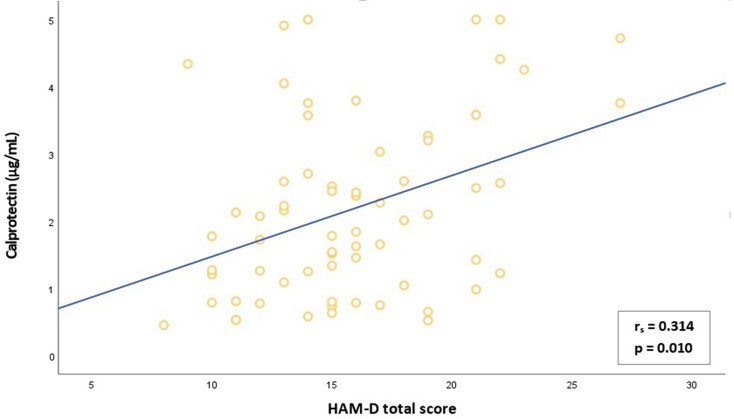
Two-dimensional scatter plot showing the correlation between serum calprotectin levels and HAM-D scores in patients with MDD. Serum calprotectin levels were positively correlated with HAM-D scores (Spearman’s rs = 0.314, *p* = 0.010)

Among patients with MDD, serum calprotectin levels were positively correlated with HAM-D scores (Spearman’s rs = 0.314, *p* = 0.010; Fig. 2).

**Fig. 3:**
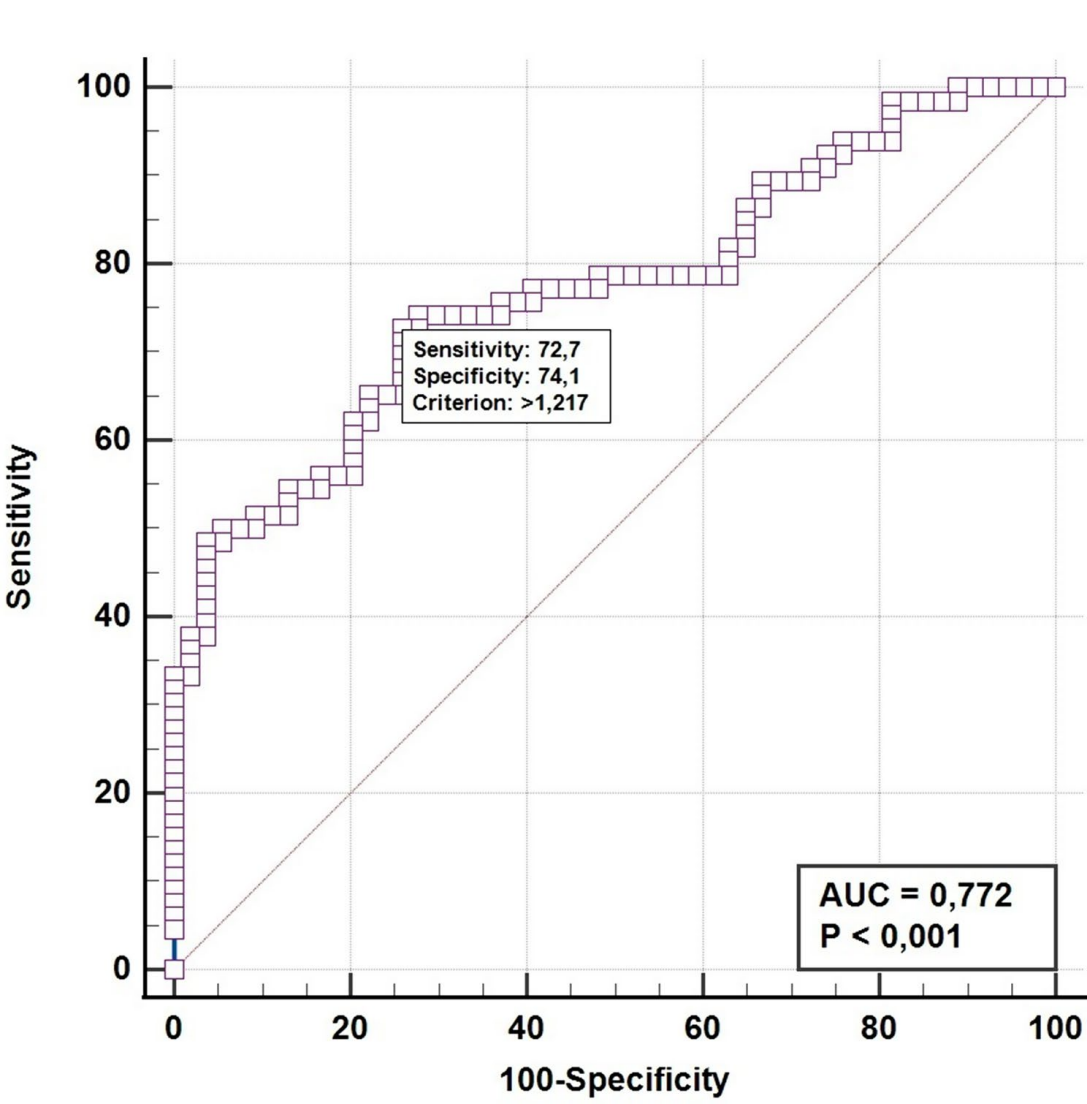
ROC curve showing the ability of serum calprotectin levels to discriminate between patients with MDD and healthy controls. At a cutoff of 1.22 µg/mL, the sensitivity and specificity were 72.7% and 74.1%, respectively (AUC = 0.772, 95% CI 0.689–0.854, *p* < 0.001)

To evaluate the ability of serum calprotectin levels to discriminate between patients with MDD and healthy controls, a receiver operating characteristic (ROC) curve was generated. The area under the curve (AUC) was 0.772 (95% CI 0.689–0.854; *p* < 0.001). At a cutoff of 1.22 µg/mL, the sensitivity and specificity were 72.7% and 74.1%, respectively (Fig. 3).

Associations of demographic, clinical, and lifestyle-related variables with serum calprotectin levels are presented in Table 2. In the MDD group, serum calprotectin levels were positively correlated with HAM-D scores (rs = 0.314, *p* = 0.010). No significant associations were observed with age, sex, suicidal ideation/behavior, smoking pack-years, alcohol use, or chronic disease status in the MDD group. In the HC group, serum calprotectin levels were not significantly associated with any of the examined variables.

**Table 2:** Associations of demographic, clinical, and lifestyle-related variables with serum calprotectin levels in patients with MDD and healthy controls

**A. Correlation analyses**		**Serum calprotectin levels**		
**Variable**			**MDD**	**HC**
Age			rs = − 0.156, *p* = 0.210	rs = − 0.083, *p* = 0.549
BMI			rs = − 0.255, *p* = 0.039	rs = − 0.144, *p* = 0.298
HAM-D score			rs = 0.314, *p* = 0.010	N/A
Smoking, pack-years			rs = − 0.144, *p* = 0.249	rs = − 0.040, *p* = 0.774
**B. Subgroup comparisons**		**Serum calprotectin levels**		
**Variable**	**Subgroup**		**MDD**	**HC**
Sex	Male vs. female		*p* = 0.263	*p* = 0.217
Alcohol use	Present vs. absent		*p* = 0.255	*p* = 0.318
Chronic disease	Present vs. absent		*p* = 0.269	*p* = 0.298
Suicidal ideation/behavior	Present vs. absent		*p* = 0.56	N/A

HC: healthy controls; MDD: major depressive disorder; BMI: body mass index; HAM-D: Hamilton Depression Rating Scale; rs: Spearman’s correlation coefficient; N/A: not applicable. Correlation analyses show associations between each variable and serum calprotectin levels within each group. Subgroup comparisons show differences in serum calprotectin levels between the indicated subgroups within each group. Correlation analyses were performed using Spearman’s correlation coefficient, and subgroup comparisons were performed using the Mann–Whitney U test. HAM-D score and suicidal ideation/behavior were evaluated only in patients with MDD

The S100A9 variant rs3014866 was consistent with Hardy–Weinberg equilibrium (χ² = 2.074, *p* = 0.150). The distribution of S100A9 rs3014866 genotypes and alleles, as well as genotype distributions under the tested inheritance models, did not differ significantly between the MDD and HC groups. In the codominant model, CC, CT, and TT genotype frequencies were 30.3%, 57.6%, and 12.1% in the MDD group and 33.3%, 57.4%, and 9.3% in the HC group, respectively (*p* = 0.856). Allele frequencies were also comparable between groups (C allele: 59.1% vs. 62.0%; T allele: 40.9% vs. 38.0%; *p* = 0.643). No significant differences were observed between the MDD and HC groups under the dominant model (CC vs. CT + TT; *p* = 0.723), recessive model (TT vs. CC + CT; *p* = 0.616), or overdominant model (CT vs. CC + TT; *p* = 0.985) (Table 3).

Serum calprotectin levels were also compared across S100A9 rs3014866 genotype groups under different inheritance models. No significant differences were observed in serum calprotectin levels in the codominant model (CC, CT, and TT: 1.65 ± 1.04, 1.66 ± 1.28, and 1.74 ± 1.24 µg/mL, respectively; *p* = 0.813), dominant model (CC vs. CT + TT; *p* = 0.676), recessive model (TT vs. CC + CT; *p* = 0.707), or overdominant model (CT vs. CC + TT; *p* = 0.529) (Table 4).

**Table 3:** Distribution of S100A9 rs3014866 genotypes, alleles, and inheritance models in patients with MDD and healthy controls

Genetic model/comparison	MDD	HC	*p*
**Codominant model**			0.856
CC, n (%)	20 (30.3)	18 (33.3)	
CT, n (%)	38 (57.6)	31 (57.4)	
TT, n (%)	8 (12.1)	5 (9.3)	
**Allelic model**			0.643
C allele, n (%)	78 (59.1)	67 (62.0)	
T allele, n (%)	54 (40.9)	41 (38.0)	
**Dominant model**			0.723
CC, n (%)	20 (30.3)	18 (33.3)	
CT + TT, n (%)	46 (69.7)	36 (66.7)	
**Recessive model**			0.616
TT, n (%)	8 (12.1)	5 (9.3)	
CC + CT, n (%)	58 (87.9)	49 (90.7)	
**Overdominant model**			0.985
CT, n (%)	38 (57.6)	31 (57.4)	
CC + TT, n (%)	28 (42.4)	23 (42.6)	

HC: healthy controls; MDD: major depressive disorder. Values are presented as n (%). p values were calculated using the chi-square test. Percentages for genotype and genetic model comparisons were calculated within each diagnostic group, whereas allele percentages were calculated based on the total number of alleles in each group. The dominant model compared T-allele carriers with CC homozygotes, the recessive model compared TT homozygotes with C-allele carriers, and the overdominant model compared heterozygotes with combined homozygous genotype groups

**Table 4:** Association of S100A9 rs3014866 genetic models with serum calprotectin levels

Genetic model	Group	Serum calprotectin, µg/mL, mean ± SD	*p*
Codominant model	CC	1.65 ± 1.04	0.813
	CT	1.66 ± 1.28	
	TT	1.74 ± 1.24	
Dominant model	CC	1.65 ± 1.04	0.676
	CT + TT	1.68 ± 1.26	
Recessive model	TT	1.74 ± 1.24	0.707
	CC + CT	1.66 ± 1.19	
Overdominant model	CT	1.66 ± 1.28	0.529
	CC + TT	1.67 ± 1.08	

Values are presented as mean ± standard deviation. Analyses were performed in the total analytic sample. Serum calprotectin levels were compared within each genetic model, with p values reported once for each model. The dominant, recessive, and overdominant models were defined as CC vs. CT + TT, TT vs. CC + CT, and CT vs. CC + TT, respectively

## Discussion

MDD is increasingly recognized as a condition that involves a low-grade inflammatory component, rather than being fully explained by monoaminergic changes alone [19]. Evidence suggests a bidirectional link between inflammatory markers and depressive symptoms. Studies consistently link depression to various immune indicators, including cytokines, acute-phase proteins, and hematological indices such as the neutrophil-to-lymphocyte ratio [20–22]. Inflammatory markers also appear to be linked not only to the occurrence of MDD but also to more severe clinical symptoms [23]. These observations suggest that proinflammatory processes may not simply be a consequence of depression but could participate in a feedback loop that increases vulnerability to MDD [24, 25]. Inflammation acts as a modifier that promotes treatment resistance and poor prognosis, and such inflammatory markers may be valuable for risk stratification and more personalized intervention strategies [23, 25]. Within this framework, neutrophil-derived mediators, such as calprotectin, may serve as biologically relevant markers in the pathophysiology of MDD.

Calprotectin (S100A8/A9) is mainly a neutrophil-derived alarmin released during phagocyte activation [26]. As an alarmin, it acts as an endogenous danger signal, or damage-associated molecular pattern, produced during cellular stress or injury to alert the innate immune system [10, 27]. By engaging pattern recognition receptors on innate immune cells, including Toll-like receptor 4 and the receptor for advanced glycation end products, calprotectin activates nuclear factor κB signaling [28, 29]. This pathway promotes leukocyte recruitment and the production of proinflammatory cytokines and chemokines, thereby sustaining systemic inflammation in which serum calprotectin acts as a positive acute-phase reactant [8, 29, 30]. Serum calprotectin levels mirror disease activity in chronic inflammatory conditions such as rheumatoid arthritis, axial spondyloarthritis, and inflammatory bowel disease, and decrease as systemic inflammation subsides; therefore, it has been proposed as a dynamic inflammatory biomarker for monitoring treatment response and relapse [31, 32]. Since monocytes and macrophages also produce calprotectin, it can be detected in serum, feces, urine, cerebrospinal fluid, saliva, and synovial fluid [33–36]. Elevated serum or tissue calprotectin has also been observed outside the gut in conditions such as psoriasis, rheumatoid arthritis, systemic lupus erythematosus, ankylosing spondylitis, periodontitis, and various cancers [32, 37–42]. Matrix-specific patterns are well described; for example, fecal calprotectin increases in colorectal cancer, inflammatory bowel disease, and bacterial gastrointestinal infections [43–45]. Taken together, these findings suggest that calprotectin could be a potential biomarker for MDD, especially in longitudinal studies that monitor changes in inflammatory burden over time [46].

Historically, most research has centered on fecal calprotectin, which is found at concentrations much higher than in plasma and remains relatively stable [47]. These properties have established fecal calprotectin as a sensitive and specific marker of gut mucosal neutrophilic inflammation and as a standard tool for diagnosing and monitoring inflammatory bowel disease [48, 49]. However, data on calprotectin in MDD and other psychiatric disorders are limited. So far, most research has evaluated depressive symptoms in patients with inflammatory bowel disease and has concentrated on fecal rather than serum calprotectin measurements [50–53]. However, fecal calprotectin primarily reflects local intestinal immune activity rather than systemic inflammation [54, 55]. For disorders characterized by low-grade systemic inflammation rather than overt intestinal pathology, such as MDD, measuring serum calprotectin provides both conceptual and practical advantages [56, 57]. Although fecal calprotectin is highly effective at detecting gastrointestinal pathology, its levels are affected by factors such as age, diet, lifestyle, mucosal immune changes, and medication use [58, 59]. It shows day-to-day and diurnal variability, often requiring repeated testing in the management of inflammatory bowel disease, which makes it less suitable for assessing systemic inflammatory activity [58–60]. Therefore, while fecal calprotectin is excellent for detecting intestinal pathology, it is not the most informative marker for systemic inflammatory activity.

In our study, serum calprotectin levels were significantly higher in patients with MDD than in healthy controls. However, findings from studies in patients with inflammatory bowel disease are mixed. For example, one study reported no difference in fecal calprotectin levels between IBD patients with and without comorbid MDD [61]. In contrast, another study of patients with inflammatory bowel disease found higher fecal calprotectin levels in those with more severe depressive symptoms and suggested that fecal calprotectin may serve as a potential screening marker for depression in this population [50]. Our observation of increased serum calprotectin in MDD extends this evidence to individuals without autoimmune or gastrointestinal disease and suggests that calprotectin-mediated immune activation may be relevant to the pathophysiology of MDD. Several factors may help explain these conflicting findings. Some studies have found no correlation between serum and fecal calprotectin [62]. This lack of correlation is consistent with the idea that markers derived from different biological compartments reflect distinct aspects of immune activation. Differences in sample size, clinical setting, patient characteristics, and overall study design may also contribute to the heterogeneous associations between calprotectin and MDD reported in the literature.

In a post-mortem study, increased microglial calprotectin immunoreactivity was observed in the brains of individuals with severe mental disorders, including major depression. This increase was interpreted as evidence of inflammatory processes contributing to their pathophysiology [63]. Peripheral proinflammatory cytokines can reach the brain and activate glial cells, leading to neuroinflammation and changes in mood and emotion [64]. In this context, our observation of higher serum calprotectin levels in patients with MDD may be consistent with a link between peripheral immune activation and central inflammatory changes.

In preclinical studies, inhibition of S100A9-driven macrophage and microglial activation has been shown to reduce depression-like behavior in rats [65]. In systemic lupus erythematosus, patients with depressive symptoms have been reported to exhibit higher serum calprotectin (S100A8/A9) levels than those without depressive symptoms [66]. In a study of peripheral blood mononuclear cells, S100A8 expression was higher in both untreated patients with MDD and SSRI-treated patients with MDD than in healthy volunteers, whereas S100A9 expression did not differ between groups [67]. This finding suggests that antidepressant exposure should be considered when interpreting S100A8/A9-related inflammatory findings. In the present study, however, all patients with MDD were newly diagnosed and had not received any antidepressant or other psychotropic treatment prior to enrollment and blood sampling. Therefore, antidepressant medication use is unlikely to have confounded the observed serum calprotectin findings. Nevertheless, our results may not be directly generalizable to patients with MDD who are receiving antidepressant treatment. Although S100A8 and S100A9 can circulate independently, they predominantly form a stable S100A8/A9 heterodimer, which is the main biologically active form in inflammatory contexts; therefore, many proinflammatory effects are attributed to the calprotectin complex rather than to the individual subunits [68, 69]. Collectively, these experimental and clinical observations implicate calprotectin as a plausible mediator of inflammatory mechanisms relevant to depression. Consistent with this, we observed higher serum calprotectin levels in the MDD group compared with the HC group. However, the distribution of the S100A9 rs3014866 polymorphism did not differ between groups. This finding may indicate that this variant does not substantially influence S100A9 production or calprotectin formation, or that any such effect was too subtle to be detected in our sample.

Several studies involving psychiatric populations, such as MDD, schizophrenia, and anorexia nervosa, have reported elevated fecal calprotectin levels [50, 70, 71]. However, most of these studies have relied on fecal measurements, and there are still very few that have examined serum calprotectin in patients with psychiatric disorders.

Evidence on the relationship between depression severity and calprotectin is mixed. One study did not find a correlation between serum calprotectin levels and depression scores, whereas another reported that higher calprotectin levels were associated with more severe depressive symptoms [50, 72]. Some studies have shown that levels of inflammatory markers, such as T helper 17 (Th17)-related cytokines, IL-6, and TNF-α, increase significantly with greater depression severity [73, 74]. However, other studies have reported no clear association between these biomarkers and the severity of depressive symptoms, which may indicate that inflammatory processes are more relevant to the onset or presence of depression than to its severity [75, 76]. Overall, these findings suggest that the relationship between inflammation and depression may not be uniform across individuals, consistent with underlying biological heterogeneity.

Findings regarding suicidal ideation and suicidal behavior have also been inconsistent. In one study, calprotectin levels were positively associated with suicidal ideation, whereas another found no correlation between serum calprotectin levels and suicidal ideation [50, 77]. In our sample, serum calprotectin was not significantly associated with suicidality, which is consistent with reports that have not confirmed clear links between inflammatory markers and suicidal outcomes. Nonetheless, this result should be interpreted with caution in view of the modest sample size, the cross-sectional design, and variability in the assessment of suicidal thoughts and behaviors. Overall, findings on immune markers in suicidality remain inconsistent, with some studies reporting elevated levels of C-reactive protein and selected cytokines, such as interleukin-6 and tumor necrosis factor-α, among individuals with suicidal ideation or behavior [78–80]. In contrast, other studies report different or absent cytokine changes, suggesting a heterogeneous inflammatory profile rather than a single, consistent pattern [81, 82]. Overall, our data do not support a specific link between calprotectin and suicidality in MDD, and further research with larger samples and longitudinal studies is needed to clarify this relationship.

### Strengths and limitations

This is, to our knowledge, the first study to investigate serum calprotectin levels in patients with MDD compared with healthy controls. Assessing both serum calprotectin and the S100A9 rs3014866 polymorphism provides initial insights into the protein and genetic factors related to calprotectin in MDD. Additionally, it is the first to explore this variant in relation to MDD and serum calprotectin.

The cross-sectional design limits our ability to draw firm conclusions about causal or temporal relationships between serum calprotectin levels and MDD. Furthermore, because only one SNP and a single inflammatory marker were assessed, the findings may not fully reflect the broader genetic and inflammatory profile associated with MDD. In addition, although rs3014866 has been examined in several inflammation-related conditions, evidence regarding its direct influence on S100A9 transcription, protein expression, or circulating S100A8/A9 calprotectin levels remains limited. Because the a priori power analysis was based on a medium effect size, the study may have had limited sensitivity to detect smaller effects, particularly in the S100A9 rs3014866 genetic analyses. Therefore, the null findings regarding this polymorphism should be interpreted cautiously and confirmed in larger samples.

## Conclusions

In this study, serum calprotectin levels were higher in patients with MDD than in healthy controls, and elevated calprotectin levels were associated with more severe depressive symptoms. These findings suggest that serum calprotectin may represent an inflammation-related marker associated with MDD. However, because of the cross-sectional design, no causal conclusions can be drawn. No associations were observed between serum calprotectin and suicidal ideation or suicidal behavior. The distribution of the S100A9 rs3014866 variant was similar between patients and controls, and this polymorphism was not associated with MDD or serum calprotectin levels. Serum calprotectin may therefore merit further evaluation as a potential inflammation-related stratification marker in future longitudinal and interventional studies.

## Data Availability

The datasets used and/or analyzed during the current study are available from the corresponding author upon reasonable request.

## Abbreviations

ANOVA: Analysis of variance
AUC: Area under the curve
BMI: Body mass index
DSM-5-TR: Diagnostic and Statistical Manual of Mental Disorders, Fifth Edition, Text Revision
HAM-D: Hamilton Depression Rating Scale
HC: Healthy controls
IL-1RA: Interleukin-1 receptor antagonist
IL-6: Interleukin-6
MDD: Major depressive disorder
MRP8/14: Myeloid-related protein 8/14
PCR: Polymerase chain reaction
ROC: Receiver operating characteristic
SCID: Structured Clinical Interview for DSM Disorders
SNP: Single-nucleotide polymorphism
TNF-α: Tumor necrosis factor-alpha
Th17: T helper 17
